# Management of Localized Muscle-Invasive Bladder Cancer from a Multidisciplinary Perspective: Current Position of the Spanish Oncology Genitourinary (SOGUG) Working Group

**DOI:** 10.3390/curroncol28060428

**Published:** 2021-12-03

**Authors:** Antonio Gómez Caamaño, Ana M. García Vicente, Pablo Maroto, Alfredo Rodríguez Antolín, Julián Sanz, María Almudena Vera González, Miguel Ángel Climent, on behalf of the Spanish Oncology Genitourinary (SOGUG) Multisiciplinary Working Group

**Affiliations:** 1 Department of Radiation Oncology, Hospital Clínico Universitario de Santiago, 15706 Santiago de Compostela, Spain; 2 Nuclear Medicine Department, University General Hospital, 13005 Ciudad Real, Spain; angarvice@yahoo.es; 3 Hospital Sant Pau, 08041 Barcelona, Spain; jmaroto@santpau.cat; 4 Urology Service, Hospital Universitario 12 de Octubre, 28041 Madrid, Spain; arantolin@yahoo.es; 5 Clínica Universidad de Navarra, 31008 Pamplona, Spain; jsanzo@unav.es; 6 Radiology Department, Hospital Clínico Universitario de Valencia, 46010 Valencia, Spain; almuverag@gmail.com; 7 Medical Oncology Service, Fundación Instituto Valenciano de Oncología, 46009 Valencia, Spain; macliment@fivo.org

**Keywords:** muscle-invasive bladder cancer, neoadjuvant chemotherapy, molecular subtypes, bladder preservation, radical cystectomy, immunotherapy, checkpoint inhibitors

## Abstract

This review presents challenges and recommendations on different aspects related to the management of patients with localized muscle-invasive bladder cancer (MIBC), which were discussed by a group of experts of a Spanish Oncology Genitourinary (SOGUG) Working Group within the framework of the Genitourinary Alliance project (12GU). It is necessary to clearly define which patients are candidates for radical cystectomy and which are candidates for undergoing bladder-sparing procedures. In older patients, it is necessary to include a geriatric assessment and evaluation of comorbidities. The pathological report should include a classification of the histopathological variant of MIBC, particularly the identification of subtypes with prognostic, molecular and therapeutic implications. Improvement of clinical staging, better definition of prognostic groups based on molecular subtypes, and identification of biomarkers potentially associated with maximum benefit from neoadjuvant chemotherapy are areas for further research. A current challenge in the management of MIBC is improving the selection of patients likely to be candidates for immunotherapy with checkpoint inhibitors in the neoadjuvant setting. Optimization of FDG-PET/CT reliability in staging of MIBC and the selection of patients is necessary, as well as the design of prospective studies aimed to compare the value of different imaging techniques in parallel.

## 1. Introduction

Bladder cancer is the most common malignancy of the urinary system, with an estimated 600,000 new cases and over 200,000 deaths worldwide annually [1]. Nearly 70% of new bladder cancer diagnoses are early stage, and have not yet invaded the muscle layer, whereas the remaining 30% of patients have muscle-invasive bladder cancer (MIBC), including cancer involving the muscularis propria (T2), perivesical tissue (T3) or adjacent pelvic organs/structures (T4) [2]. The treatment of MIBC is complex and is based on a multidisciplinary collaboration between surgery, radiotherapy and medical oncology teams. Radical cystectomy with lymph node dissection and systemic cisplatin-based combination chemotherapy either before or after radical cystectomy has been considered the standard treatment approach in MIBC. However, as many patients are unfit for surgery or are cisplatin-ineligible, bladder-sparing strategies are increasingly recognized as optimal treatment options in selected patients that can be presented at the time of diagnosis [3,4]. Moreover, apart from chemotherapy, radiation and immunologic therapeutic options, especially checkpoint inhibitors are selective strategies being incorporated in the therapeutic landscape of MIBC [5,6].

The Genito Urinary Alliance project (12GU) was designed as a space for the integration of innovation progress in the management of patients with bladder cancer. For this purpose, expert members of the Spanish Oncology Genitourinary (SOGUG) Multidisciplinary Working Group discussed some controversial and debatable topics of the current knowledge and approach in the care of patients with localized MIBC. The aim of the project was to summarize practical recommendations on some particular aspects of localized MIBC, including molecular-based analysis for the classification of urothelial carcinoma, bladder-sparing approaches and new molecular classifications, integral evaluation of candidates for radical cystectomy, use of neoadjuvant therapy and integration of immunotherapy, and the role of imaging techniques in staging, assessment of treatment response and follow-up. Challenges and recommendations were reached by agreement of all participants to be applicable in clinical practice to facilitate shared decision making for individual patients diagnosed with MIBC.

## 2. Subclassification of Urothelial Carcinoma in Different Molecular Groups

The Service of Pathology is currently shifting to play an active role in driving forward ‘personalized predictive pathology’, moving beyond diagnosis and mere classification and subclassification of diseases to become the field responsible for providing personalized medical information [7]. In this context, besides morphology, margins and staging, pathologists select the tumor tissue, microdissect and enrich the tumor, test for mutations at the molecular level including genotyping, now using next-generation sequencing (NGS), real-time polymerase chain reaction (RT-PCR), in situ hybridization and proteomic by immunohistochemistry, all of which allow combination of morphological, molecular and clinical data to provide the oncologist with a comprehensive pathological report.

The correct morphological classification of urothelial cancer is complex, because there is great tumor heterogeneity and a wide variety of histopathological patterns [8]. The current World Health Organization (WHO) classifications clarify terminological issues and provide better definition criteria. Histological variants include: urothelial carcinoma with divergent differentiation, and nested, microcystic, micropapillary, lymphoepithelioma-like, plasmacytoid/signet ring cell/diffuse, giant cell, lipid-rich, clear cell (glycogen-rich), and poorly differentiated urothelial carcinomas. The urothelial carcinoma with divergent differentiation includes squamous, glandular, trophoblastic and other types of differentiation [8].

The College of American Pathologists recommends that the percentage of morphological subtype differentiation should be specified in the pathology report [9]. Experimental studies using cytogenetic, molecular genetics and immunohistochemical methods have provided new insights into the molecular mechanisms and pathways involved in bladder cancer. Frequent genetic abnormalities include *CYP1A* and *GSTM1* polymorphisms, methylation of GpC sites, and mutations with *FGRF3* and *p53* as the most common [10]. On the other hand, carcinogenicity may result in pan-urothelial and multifocality of urothelial cancer. It is also accepted that human bladder cancer develops via two distinct, but sometimes overlapping pathways, papillary and non-papillary. In the papillary pathway of superficial or low-grade lesions (80% of cases), there is activation of proliferative factors (FGFR3 and HRAS), whereas in the non-papillary pathway (20% of cases), including carcinoma in situ (CIS) and infiltrating carcinomas, there is a loss of p53 or RB1 function. In 15% of cases with genetic instability and involvement of the RB1/p53 pathway, severe intraurothelial dysplasia/CIS (HGIN) developing in bladder mucosa adjacent to a low-grade papillary tumor may be responsible for switching the pathway and progression of some low-grade papillary tumors to high-grade invasive cancers [11].

Distinct basal and luminal subtypes of MIBC have been identified, with immunohistochemical markers for basal cells (CD44, CK5), intermediate cells (PPARG, GATA3, CK18) and umbrella cells (CK20, uroplakin) [12]. Two molecular subtypes were validated by assessing the genomic expression profiles of bladder cancer in frozen tumor samples from three cohorts (MD Anderson hospital, Lund, the Cancer Genome Atlas, with 132, 132, and 408 cases, respectively) included in a meta-analysis. It was found that the immunohistochemical expression of luminal (GATA3) and basal (KRT5/6) was sufficient to identify bladder cancer molecular subtypes with >90% accuracy [13]. In a model of five mRNA-based expression clusters, reported in 2017, not only were the basal and luminal subtypes identified, but these subtypes were also stratified into five different categories, including the basal-squamous subtype (35%), the luminal-papillary subtype (35%), the luminal-infiltrated subtype (19%), the luminal subtype (6%), and the neuronal subtype (5%) [14]. Each subtype was further characterized by histological findings, biomarker expression, likelihood of response [15], and suggested treatments. Finally, in 2020, a single consensus set of molecular subtypes was defined based on previously published MIBC classifications, which converged on six biologically relevant molecular classes, labeled as luminal papillary (LumP), luminal nonspecified (LumNS), luminal unstable (LumU), stroma-rich, basal/squamous (Ba/Sq), and neuroendocrine-like (NE-like) [16]. Each consensus class has distinct differentiation patterns, oncogenic mechanisms, tumor microenvironments, and histological and clinical associations [16].

**Figure 1:**
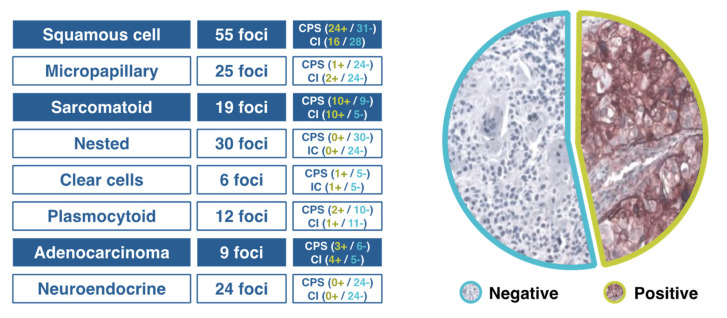
Results of a Spanish study of PD-L1 expression in different variants of urothelial cancer.

In patients with advanced urothelial carcinoma, promising clinical activity has been shown for antibodies targeting the programmed cell death-1 (PD-1)/PD-ligand 1 (PD-1/PD-L1) checkpoint, but when different algorithms have been used to assess high vs. low/negative PD-L1 expression, the extent of concordance of the available PD-L1 immunohistochemical (IHC) assays has been poorly defined. The comparison of technical performance and characteristics of different assays and algorithms will allow a more accurate interpretation of outcomes associated with anti–PD-1/PD-L1 therapies in patients with urothelial cancer. When four commercially available PD-L1 assays (PD-L1 IHC 28–8 pharmDx, PD-L1 IHC 22C3 pharmDx), SP142, and SP263) were used in the analysis of biopsy samples from 335 tumors, in all assays except for SP142, the analytical concordance was high for both tumor cells and the proportion of tumor infiltrating area with PD-L1 staining [17]. In a Spanish multicenter study aimed at assessing PD-L1 expression in different tumor variants using CPS and SP142 assays, tumors with squamous cell or sarcomatoid differentiation and adenocarcinomas showed a high PD-L1 expression (accounting for 50% in some cases), whereas PD-L1 expression was almost absent in micropapillary, nested, plasmacytoid, clear cells and neuroendocrine variants (Figure 1). Tumors with squamous cell differentiation mostly belong to the basal molecular subtype, whereas micropapillary, nested and plasmacytoid variants are included in the luminal molecular subtype [18].

On the other hand, it has been reported that sarcomatoid carcinoma entails enrichment mutations of TP53, RB1, and PIK3CA, as well as mutagenesis signature 1, in the progression of conventional urothelial carcinoma of the basal subtype. This process is driven by dysregulation of the EMT network (epithelial-to-mesenchymal transition) and characterized by increased immune infiltration with PD-L1 overexpression [19]. In a series of 27 tumor types or subtypes among patients who received PD-1 checkpoint inhibitors, tumor mutation burden (the total number of mutations per coding area of a tumor genome) was found to be correlated with objective response [20], although in the case of urothelial cancer, analysis by molecular subtypes was not performed. It has also been found that some patients treated withanti-PD1/PDL1 monotherapy present a hyper-response with a greatly accelerated rate of tumor growth and clinical deterioration [21]. Hyper-progressors harbored MDM2/4 or EGFR alterations, suggesting the need for caution in the presence of these genomic profiles [21]. At the present time, evidence of the use of other biomarkers as predictors of response to immunotherapy with PD-1 checkpoint inhibitors in the different molecular subtypes of MIBC is lacking.

### Challenges and Recommendations

- To establish a correct classification of histopathological subtypes of MIBC, particularly the identification of subtypes with prognostic, molecular and therapeutic implications.
- Immunohistochemical techniques should be used in order to distinguish between basal and luminal subtypes.
- In relation to the molecular classification of MIBC, luminal subtypes as well as stroma-rich and neuroendocrine-like ones are difficult to characterize.
- PD-L1 is still the most relevant biomarker in urothelial cancer, and the association of its expression with some variants merits further investigation.

## 3. Integrated Assessment of Patient Candidates for Radical Cystectomy

Radical cystectomy is an aggressive surgical procedure, with a high rate of complications and perioperative mortality. The 30-day and 3-month treatment associated mortality for radical cystectomy was 3.1% and 8.3% in a cohort of 398 patients with MIBC in the Yorkshire region [22]. In a nationwide population-based study in Spain of 12,154 patients from 196 hospitals, the 90-day mortality rate was 6.5% [23]. Surgical morbidity following radical cystectomy is also significant, with the rate of overall complications ranging between 27% and 64% [24]. Other factors to be considered include the high mean age of the patients, class II–III of the American Society of Anesthesiologists (ASA) physical status classification in more than 50% of patients, poor previous nutritional status in some patients, and the fact that enhanced recovery after surgical (ERAS) protocols are not universally standardized, difficult to apply and show variable results. Data of 122 patients with MIBC undergoing radical cystectomy between January 2012 and December 2017 in a tertiary care hospital in Madrid, Spain, showed early postoperative complications in 45% of patients (17% requiring any type of surgical reoperation), 90-day complications in 59% of patients (Clavien-Dindo grades II and III in 49% and 45% of cases, respectively), and late complication in 38%.

In relation to candidates for radical cystectomy, there has been a progressive increase in the number of older and fragile patients. In a review of the results of radical cystectomy in 111 patients with a median age of 82.2 years, the early and late complication rates were 50.4% and 32%, respectively; moreover, 12.6% required surgical reintervention and 7.2% died in the immediate postoperative period [25]. At follow-up (mean 18 months), 66 patients had died, 52 of them due to the tumor, and 40 patients (38.8%) showed tumor progression after six chemotherapy cycles [25]. Treatment decisions in old patients with MIBC are a difficult clinical challenge today and alternatives such as tri-modality therapy need to be considered within a multidisciplinary approach.

Surgical outcomes improved in association with centralization of care with advantages at high-volume centers (mortality rate decreases from 6.5% to 3.3% in centers with an annual rate of radical cystectomy >38 cases) [23,26], the surgeon’s experience, the availability of clinical pathways for postoperative management, and early suspicion and management of complications (potent interventional radiology service).

There is little information on ERAS programs in patients with MIBC undergoing radical cystectomy. However, in relation to postoperative complications, use of analgesics, length of stay in the intermediate care unit, and quality of life, data of a prospective randomized study showed significant benefits for ERAS versus a conservative regimen [27]. However, in an analysis of data of 277 patients prospectively recruited in 11 Spanish hospitals, complications, length of stay and 90-day mortality were not modified by the introduction of ERAS, although the risk of having any complication decreases for patients having more than 15 components of ERAS protocol adopted [28].

The implementation of a multimodal perioperative protocol with different preoperative, intraoperative, and postoperative measures to reduce complications, hospital stay, readmissions and to improve the patient’s quality of life is recommended. Some of the main measures may include the following: to perform minimally invasive surgery, no oral bowel preparation, no nasogastric tube, no drains, no morphics, restriction of fluids, initiation of oral tolerance after 6 h, and mobilization of the patient after 8 h. Optimization of the patient’s preoperative condition is based on “prehabilitation” measures, such as complete an accessible oral and written information, management of urostomies/catheterizations, change of lifestyle habits (smoking, physical exercise), anemia correction, nutritional assessment and support, carbohydrate load, preoperative fasting (clear fluids to 2 h, solids to 6 h), and no benzodiazepines among preanesthetic drugs. Other measures include assessment of the fragility of patients using the Clinical Frailty Scale (CFS) as a way to summarize the overall level of fitness or frailty of an older adult [29], preoperative physiotherapy, and identification of patients who require specialized previous evaluation, such as those at high cardiovascular risk, high nutritional risk, high transfusional risk (serum hemoglobin level 6–10 g/dL), and patients with poorly controlled hypertension and diabetes mellitus.

### Challenges and Recommendations

- In patients older than 80 years, it is necessary to include a geriatric assessment, and evaluation of comorbidities and frailty. The presence of comorbid diseases may be more relevant than the age per se.
- In the framework of ERAS protocols, although postoperative measures are the most relevant, preoperative assessment of candidates for radical cystectomy by the corresponding specialists is recommended, as well as the adequate preparation of patients regarding his/her condition and imitations after surgery.
- Outcomes could be improved by centralization of care in high-volume centers with experienced multidisciplinary teams.

## 4. Bladder Preservation and New Molecular Classifications

Bladder preservation strategies aim to achieve a maximum control of cancer with the preservation of bladder function and subsequent benefits on quality of life and survival. In selected patients with MIBC, it has been shown that chemoradiation following an aggressive transurethral (TUR) resection of bladder tumor (TURBT) can be an effective and safe alternative treatment to radical cystectomy [30]. Although biomarker studies based on TURBT surgical specimens indicate that a favorable response to organ-preservation treatment using radiation therapy or chemotherapy regimens can be obtained in some subsets of patients, these observations should be validated in prospective studies.

Evidence of selective bladder preservation is based on two single-center studies conducted at the Massachusetts General Hospital in Boston [31] and the University of Erlangen in Germany [32], in which similar protocols were used except for split-course radiation with induction chemoradiation (~40 Gy) or single-course radiation with full-dose chemoradiation (55–65 Gy) after maximal TURBT and before cystoscopic re-biopsy for the assessment of treatment response. Long-term results were similar in both studies with complete response rates in 70% of patients, local control in 40–60%, distant metastasis in <40%, overall survival in 25–35%, salvage cystectomy in 20–30%, and bladder preservation in 80% of survivors. The pooled results of phase II and phase III studies showed complete response in 69% of patients, and 5- and 10-year rates of 56% and 55% for cystectomy-free survival, 71% and 65% for disease-specific survival, and 57% and 36% for overall survival, respectively [30].

In relation to the advantages of synchronous chemoradiotherapy over radiotherapy alone, a phase III multicenter study of 360 patients with MIBC randomized patients to a regimen of fluorouracil and mitomycin, and whole-bladder radiotherapy or modified-volume radiotherapy, with survival-free of locoregional disease as the primary outcome [33]. Synchronous chemotherapy improved locoregional control of bladder cancer as compared to radiotherapy alone (locoregional disease-free survival at 2 years 67% vs. 54%, hazard ratio (HR) 0.68, 95% confidence interval (CI) 0.48–0.96, *p* = 0.03), invasive locoregional disease-free survival at 2 years 82% vs. 68%, HR 0.57, 95% CI 0.37–0.90, *p* = 0.01), but differences in overall survival at 5 years were not found [33]. Other phase III studies have confirmed the superiority of chemoradiotherapy as compared with radiotherapy alone [34,35]. Less toxicity has been shown with the use of gemcitabine versus cisplatin-based chemotherapy [36]. Optimizing radiotherapy strategies including dose escalation (minimum dose 60 Gy), hyperfractionated radiation (hypofractionated accelerated radiotherapy is not recommended), hyperthermia combined with radiotherapy, and centralization in specialized centers have been recommended [37,38,39,40,41].

Patient selection is a key component of bladder preservation [42]. Candidates for bladder preserving approaches are shown in Table 1.

Although both molecular biomarkers can be very useful for guiding the selection of candidates for bladder-preserving treatment approaches, successful translation of the value of these predictive and prognostic biomarkers into clinical practice is difficult [43]. Table 2 includes a description of these selected biomarkers. Overexpression of MRE11, which is a DNA repair-involved protein, has shown to be of value as a predictive biomarker of disease-specific survival after radiation or chemoradiation therapy, but failed for predicting cystectomy outcome [44,45,46].

When interpreting the evidence of bladder preservation therapy versus cystectomy in patients with MIBC, the following should be noted: (a) most data have been collected from retrospective observational and registry studies with methodological differences and selection bias; (b) the trimodal therapy for MIBC has been mostly limited to patients unfit for cystectomy because comparative phase III trials between radical cystectomy and TUR with chemoradiotherapy are lacking (in fact, a randomized phase III study comparing selective bladder preservation and radical cystectomy after neoadjuvant chemotherapy was not feasible [47]); (c) it is complex to assess surgical versus non-surgical options in the radical treatment of MIBC due to patient- and specialist-related factors; and (d) in clinical practice, bladder preservation is limited to some centers or hospitals of excellence with strongly motivated professionals regarding the effectiveness of bladder preserving options. In addition, the success of a multimodal approach requires the implementation of very strict follow-up protocols, self-motivated and disciplined patients, coordination of specialists and protocolized activities to ensure the optimization of results.

In the 2021 annual meeting of the American Society of Clinical Oncology (ASCO), data of phase II studies integrating immunotherapy in bladder preservation protocols were presented (Table 3) [48,49,50]. In general, these treatment schemes were safe and showed a high efficacy in terms of response and bladder preservation, although longer follow-up period are needed to establish the impact of this strategy on long-term survival.

### Challenges and Recommendations

- Bladder preservation may be an adequate strategy in the management of MIBC based on selection of the appropriate candidates.
- Bladder preservation does not compete with radical cystectomy, it is simply a complementary alternative.
- Bladder preservation strategies cannot be implemented in clinical practice without the presence of a urologist responsible for performing three main activities for success: maximal TUR, follow-up cystectomy and salvage cystectomy.
- Transdisciplinary collaboration needs to be potentiated for generating, sharing, and integrating knowledge, coordination of patient’s care activities, and to converge in the research effort.

## 5. Neoadjuvant Chemotherapy and Integration of Immunotherapy

Neoadjuvant platinum-based chemotherapy provides significant benefits in terms of downstaging and oncological outcome in patients with MIBC. In a meta-analysis of 11 randomized controlled trials with 3005 patients, platinum-based combination chemotherapy, as compared to controls, showed an absolute survival benefit of 5% at 5 years [51]. Different studies have quantified the effect of different regimens of neoadjuvant chemotherapy on the tumor stage, with percentages of surgical specimens pathologically free of cancer (pT0) at the time of cystectomy between 20% and 38% [52,53,54,55,56,57] (Table 4).

Data of a retrospective cohort study of 1543 patients, who between 2000 and 2013 had been treated with neoadjuvant chemotherapy and radical cystectomy, pT0N0 and pTa/Tis/T1N0 disease was reported in 257 and 207 patients, respectively [58]. Moreover, a strong predictor of survival was the detection of non-muscle-invasive residual cancer (pTa, pTis, pT1) [58]. The incorporation of histological subtypes and molecular classifications in the selection of patients with MIBC for neoadjuvant chemotherapy, which in clinical practice is mainly based on clinical criteria, would increase the current percentage of 30–40% of patients with pT0 disease. 

Neoadjuvant chemotherapy; however, is given in only 20% of eligible patients and is still not a widely used treatment for MIBC patients. In addition, preexisting contraindications are responsible for cisplatin ineligibility in about 50% of patients and treatment with any kind of chemotherapy is refused by another subset of patients. Also, despite the efficacy of neoadjuvant chemotherapy, up to 50% of patients may present pT2 or higher high-risk residual disease, with poor prognosis. Undoubtedly, a great advance in the management of urothelial cancer has been the use of immune checkpoint inhibitors (programmed cell death protein-1 (PD-1) and programmed death ligand-1 (PD-L1) agents). Short courses of immunotherapy integrated in the management of non-metastatic MIBC may become a useful approach for neoadjuvant treatment.

Neoadjuvant treatment with PD-1 or PD-L1 has recently shown promising results, with pT0 rates similar to those reported with neoadjuvant chemotherapy. The ABACUS trial (a single-arm phase II study) in which 95 patients with MIBC received two cycles of atezolizumab prior to cystectomy showed a complete response rate of 31% (95% CI 21% to 41%) [59]. The presence of stromal factors, including fibroblast activation protein and transforming growth factor-β were associated with resistance, as opposed to predominant expression of genes related to tissue repair related to responding tumors [59]. In the PURE-01 study, which was a single-arm open-label phase II study of neoadjuvant treatment with pembrolizumab in MIBC patients who were candidates for radical cystectomy, a complete response with pT0 was obtained in 42% of patients (21/50), and downstaging to pT < 2 in 54% (27/50) [60]. In the updated results of this study in the subpopulation of 34 MIBC patients with predominant variant histology (defined as involving >50% of the tumor specimens, are typically excluded from clinical trials, and for these patients, the efficacy of standard chemotherapy is limited), neoadjuvant treatment with pembrolizumab achieved pT0 in 37% (95% CI 28–46%) and the pT ≤ 1 in 55% (95% CI: 46–65%) [61]. Neoadjuvant pembrolizumab was also suitable for patients with squamous-cell carcinoma (SCC) and lymphoepithelial-like (LEL) variants, as six of seven patients with SCC had downstaging to pT ≤ 1, with one pT0, and two of three patients with LEL had a pT0 response [61].

Recently, an interferon (INF)-gamma immune signature was used for selecting patients with MIBC to participate in a prospective randomized phase II study (DUTRENEO trial) to assess a neoadjuvant treatment with durvalumab and tremelimumab as compared to chemotherapy [62]. Nanostring technology was used to establish a tumor immune score according to which patients with cT2-T4a, N ≤ 1, M0 urothelial MIBC eligible for cisplatin therapy and candidates for radical cystectomy were classified into “cold” and “hot”. Patients in the “cold” group were assigned to standard chemotherapy with cisplatin (n = 16), whereas patients in the “hot” group were randomized (1:1 ratio) to cisplatin-based chemotherapy (n = 22) or treatment with durvalumab and tremelimumab (n = 23). In “cold” tumors, complete response (pT0) was observed in 68.8% of patients, pathological partial response in 6.3%, and downstaging in 75%. In “hot” tumors treated with durvalumab and tremelimumab, complete response occurred in 34.8% of patients, pathological partial response in 21.7%, downstaging in 56.5%, and progressive disease in 4.3% of the patients. Results obtained in “hot” tumors treated with cisplatin-based chemotherapy were similar. This study showed that the combination of durvalumab and tremelimumab was safe and active in MIBC patients in the neoadjuvant setting. Nevertheless, prospective stratification by a pro-inflammatory IFN-γ signature failed to select patients more likely to benefit from neoadjuvant immuno-oncological drugs vs. standard chemotherapy in this context [62].

In relation to postoperative adjuvant therapies, patients initially treated with radical surgery and at high risk of recurrence according to pathological staging (pT3-4, pN+) are potential candidates for adjuvant treatment with chemotherapy [63] or immunotherapy (if they had received neoadjuvant chemotherapy) [64]. Adjuvant radiation therapy may also be considered in patients with positive surgical margins due to the high risk of loco-regional recurrence [65].

### Challenges and Recommendations

- Improvement of clinical staging, better definition of prognostic groups based on molecular subtypes, and identification of biomarkers potentially associated with maximum benefit from neoadjuvant chemotherapy are areas for further research.
- A current challenge in the management of MIBC is to improve the selection of patients likely to be candidates for immunotherapy with checkpoint inhibitors in the neoadjuvant setting.
- Patients with complete response (pT0) after neoadjuvant treatment may be suitable for bladder preservation procedures.
- Neoadjuvant therapy is not a widespread practice, although patients treated with neoadjuvant chemotherapy/immunotherapy present a better condition for undergoing cystectomy.
- The implementation of multidisciplinary teams will extend the use of neoadjuvant therapy and improve outcomes.

## 6. Role of Imaging Techniques in Staging, Assessment of Response, and Follow-Up

### 6.1. Nuclear Medicine

[18F]-Fluorodeoxyglucose (FDG)-positron emission tomography (PET) with multislice helical computed tomography (CT) (F18-FDG PET/CT) is currently a standard imaging tool used in the diagnosis and control of patients with MIBC. In 2017, the guidelines of the European Association of Urology (EAU) considered FDG-PET/CT to be an imaging procedure pending evaluation, but the National Institute for Health and Care Excellence (NICE) [63] has approved the use of FDG-PET/CT in high-risk patients with muscle-invasive or non-muscle-invasive bladder cancer (pTaG3, pT1G2, pT1G3, pTis), in the presence of aggressive variants (micropapillary or nested subtypes), indeterminate findings of CT and magnetic resonance imaging (MRI), or at high risk of metastatic disease (e.g., T3b). In 2018, an expert panel on urologic imaging recommended PET/CT for staging of MIBC in the initial patient assessment [64]. In the 2020 guidelines of the National Comprehensive Cancer Network (NCCN), FDG-PET/CT is recommended for staging ≥ IIIA (T3N0) (level of evidence 2b) [65].

Clinical studies have shown that FDG-PET/CT provides important additional staging information, which influences the treatment of MIBC in 18–68% of cases due to correct overstaging as compared to conventional imaging techniques [66,67]. FDG-PET/CT shows a similar accuracy to that of MRI in N-staging, with faster whole-body acquisition times and a high accuracy in M-staging. Data of systemic reviews and meta-analyses of the diagnostic accuracy of FDG-PET/CT for preoperative lymph node staging in newly diagnosed bladder cancer patients have shown moderate sensitivities similar to MRI and high specificities [68,69,70,71] (Table 5). 

**Figure 2:**
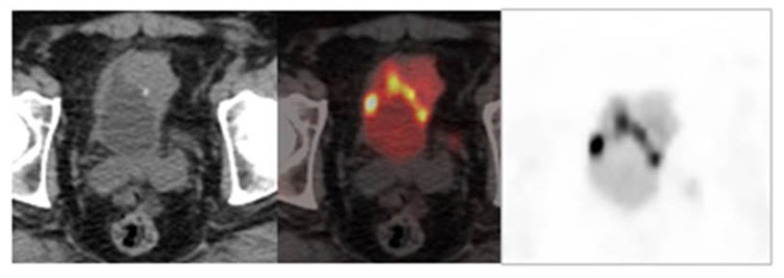
Image obtained with FDG-PET/CT after the administration of a diuretic.

On the other hand, diuretic FDG PET/CT is highly sensitive and specific and plays an important role in improving detection of the primary tumor and locoregional staging of urinary bladder tumors [72] (Figure 2).

There are no sufficiently effective techniques for assessing the response to neoadjuvant chemotherapy in MIBC patients. It has been shown that clinical endoscopic staging after neoadjuvant chemotherapy is inadequate to accurately assess for residual disease. In a study of 318 with MIBC who underwent neoadjuvant chemotherapy and radical cystectomy, biopsy performed following chemotherapy for restaging was unreliable in 50% of cases for predicting pathologic T stage at radical cystectomy [73]. In another study, of 52 patients, systematic endoscopic evaluation after neoadjuvant treatment missed nearly 30% of ≥pT2 urothelial pathology [74]. The usefulness of FDG-PET/CT in the selection of patients prior to neoadjuvant therapy and the assessment of response seems promising, but the experiences published so far are still limited to small retrospective clinical series of patients, the methodological heterogeneity of which prevents a comparison of results [75,76,77]. However, it seems that FDG-PET/MRI for preoperative staging of MIBC performs similarly to CT for the detection of the primary tumor and is more limited for the detection of lymph node status [78]. There are inconclusive data regarding the role of FDG-PET/CT in the evaluation of results of neoadjuvant treatment, definition of the time to perform an interim evaluation (whether after one, two or three chemotherapy cycles), the interval between the last cycle and FDG-PET/CT, and the interpretation of eventual response.

Standardized follow-up of MIBC is based on chest images and abdominopelvic CT or MRI scans every 6–12 months during the first 2–3 years and then at annual intervals over the first 5 years. FDG-PET/CT is not used routinely in the follow-up of patients, although it may be useful when this technique has not been previously performed or when recurrence is suspected (NCCN 2020 guidelines) [65].

#### Challenges and Recommendations

- Optimization of FDG-PET/CT reliability in staging of MIBC.
- Design of prospective studies aimed to compare the value of different radioimaging techniques in parallel, and to define the impact of FDG-PET/CT in the selection of patients.
- To determine which imaging technique is most effective for predicting response after neoadjuvant chemotherapy or a guide for endoscopic biopsy.
- To unify the methodology for acquisition and interpretation of FDG-PET/CT.
- To define predictive risk models in which FDG-PET/CT would have the highest reliability in the detection of recurrence.
- To perform prospective studies for the comparison in parallel of FDG-PET/CT with other techniques for detecting recurrence in intermediate/high-risk patients.

### 6.2. Radiology

The role of imaging techniques in the diagnosis of MIBC, in general, is neither correctly defined nor standardized. Ultrasound is a well-accepted, cost-effective, and noninvasive diagnostic method for the screening of patients with suspicion of bladder cancer, with hematuria or symptoms of the low urinary tract, although ultrasound may be inconclusive in the detection of small tumors (<1 cm), flat lesions or with atypical morphology. Contrast-enhancement ultrasound (CEUS) improves detection of lesions in patients with acute hematuria and high suspicion of bladder tumor, and is useful to differentiate clots from parietal lesions as well as tumors located on the bladder base from intravesical prostatic protrusion. However, CEUS is still not routinely used in daily practice.

The usefulness of radiological techniques for staging is related to detection of local invasion of the bladder walls, local or retroperitoneal lymph nodes, involvement of the upper urinary tract, and hematogenous metastases. CT urography is recommended by guidelines [63,65] in TNM staging due to several advantages, including assessment o extension or perivesical fat, adjacent organs and pelvic wall; evaluation of the upper urinary tract to exclude synchronic tumors (technique of choice); number and location of lymph nodes; and distant spread (chest CT). CT urography has a higher spatial resolution and faster acquisition times as compared with MRI, although limitations include use of radiation, no differentiation of the three layers (important in pT3 tumors), and difficulties in assessing changes after a surgical procedure or radiotherapy. Evaluation of lymph nodes using CT urography is based on size, shape and density of nodes.

In relation to assessment of the clinical stage using MRI, the advantages as compared with CT include better tissue resolution with the option of using diffusion MRI (diffusion-weighted MRI) and differentiation of the layers of the urinary wall. Limitations, however, include the need to have a full bladder for this examination, lower accessibility compared to CT, and the fact that use of MRI is not generalized in the clinical setting.

It is important to determine accurately whether there is infiltration of the muscularis propria (T stage) for treatment decisions in bladder cancer patients, since the therapeutic approach is based on differentiating MIBC from non-invasive muscle bladder cancer. Data provided by CT and MRI are not sufficiently accurate to establish the clinical stage and guide treatment. The Vesical Imaging-Reporting and Data System (VI-RADS) reported in 2018, a new approach to assess muscularis bladder infiltration by malignant cells [79,80]. This method combines T2-weigthed imaging (T2W1), dynamic contrast enhanced (DCE), and diffusion-weighted imaging (DWI) sequences according to which the possibilities of muscle invasion can be established. The VI-RADS scoring system is based on a 5-point scale (from 1 = very unlikely to 5 = very likely). The new VI-RADS scoring system should be further validated and evaluated in different clinical scenarios, including prospective and randomized studies [81].

#### Challenges and Recommendations

- DWI has a promising role in the assessment of response to treatment, but evidence based on prospective studies is needed.
- MRI should be optimized for the differentiation of recurrence versus inflammation and fibrosis, as well as for the assessment of lymph node status.
- The interaction of different radiological techniques in the diagnosis of MIBC should be improved.
- MRI is the technique of choice for local staging, but it needs to be widely available and reproducible among hospitals.

## 7. Conclusions

Bladder cancer is an important oncological challenge that requires an indispensable multidisciplinary approach, so that all specialties involved in the diagnosis and treatment of patients with this urothelial malignancy can provide knowledge and abilities for optimal decision making in the patient’s benefit. In many countries, radical cystectomy with neoadjuvant chemotherapy is considered the standard treatment of MIBC. However, the fact of being a treatment with a relevant risk for morbimortality and a high impact on the quality of life forces a rigorous pre-surgical evaluation, as well as the implementation of protocolized measures aimed at preventing postoperative complications. Although multimodal treatment with the intent of preserving the bladder (TURBT followed by chemoradiotherapy) has been recognized as an alternative to cystectomy in highly selected patients due to similar oncological results and an acceptable safety profile, the use of bladder-sparing procedures is very limited in clinical practice due to different barriers hindering implementation. Advances in local and systemic treatments (including integration of immunotherapy) as well as in the fields of pathology, molecular biology, and radioimaging will contribute to personalized therapeutic strategies and result in a positive impact on the process of diagnosis, tumor staging and assessment of response. Although basic and clinical fields are covered in this review, a better definition of prognostic groups based on molecular subtypes and identification of biomarkers potentially associated with maximum benefit from neoadjuvant chemotherapy and immunotherapy are areas for further research.

## Figures and Tables

**Table 1:** Characteristics of candidates for bladder preservation therapies in MIBC.

Candidates	Characteristics
Ideal candidate	T2 stage
	No hydronephrosis
	No carcinoma in situ (CIS)
	Visibly complete TURBT
	Unifocal tumor
	Good bladder function and capacity
Less than ideal candidate	T3a stage
	Incomplete TURBT
	Poor bladder function or capacity
Relative contraindications	T3b-T4a stage
	Diffuse CIS
	Lymph node positive disease
Absolute contraindications	T4b stage
	Tumor-related hydronephrosis
	Prostatic stromal invasion
	Prior pelvic radiation therapy
	Not being a candidate for chemotherapy

**Table 2:** Prognostic and predictive molecular biomarkers for the selection of candidates for bladder-sparing therapy in MIBD.

Mechanism	Biomarker	Value	Clinical Correlation
DNA repair genes	*MRE11*	Predictive	Disease-specific survival
	*ERCC1*	Prognostic	Disease-specific survival
Signal transduction genes	*EGFR*	Prognostic	Disease-specific survival
	*HER2*	Prognostic	Disease-specific survival
	*VEGF*	Prognostic	Overall survival
Immune checkpoints	PD-L1	Prognostic	Local recurrence-free survival
Molecular signatures	Hypoxia	Predictive	Local recurrence-free survival
	Immune response	Predictive	Disease-specific survival

**Table 3:** Phase II studies of immunotherapy integrated in bladder preservation protocols.

Study (Reference)	Patients	Scheme	IO	RTP	QMT	Primary Endpoint	Result
HCRN GU 16-257 [48]	76	QMT-IO	Nivolumab	No	Gem/Cis	cCR	cCR 48% BI-DFS 78% (1 year)
NCT02621151 [49]	54	IO-TUR- IO/CRTR-TUR	Pembrolizumab	64 Gy (hypo)	Gem	BI-DFS (2 years)	cCR 80% BI-DFS 88% (1 year)
Immunopreserve- SOGUG [50]	32	TUR-IO/RTP-TUR	Durvalumab + tremelimumab	64 Gy (conv)	No	cCR	cCR 78% BI-DFS 73% (1 year)

IO: immunotherapy; RTP: radiotherapy; QMT: chemotherapy; Gem: gemcitabine; cis: cisplatin; cCR: complete clinical response; BI-DFS: disease-free survival with intact bladder; TUR: transurethral resection; CRTR: chemoradiotherapy.

**Table 4:** Pathologic downstaging in surgical specimens in patients with MIBC treated with neoadjuvant chemotherapy.

First Author (Reference)	Patients No.	Regimen	Pathological Stage		
			pT0N0, %	<pT2N0, %	≥pT2, %
Grossman [52]	126	MVAC	38	44	56
Iyer [53]	154	Gemcitabine-cisplatin	21	46	56
Dash [54]	42	Gemcitabine-cisplatin	26	36	64
Yeshchina [55]	37	Gemcitabine-cisplatin	25	50	50
Choueiri [56]	39	ddMVAC	26	49	51
Plimack [57]	44	ddMVAC	38	53	47

MVAC: methotrexate, vinblastine, doxorubicin, and cisplatin; dd: dose-dense.

**Table 5:** Diagnostic accuracy of imaging techniques for initial lymph node staging in bladder cancer patients.

First Author (Reference)	Technique	Number of Studies (Number of Patients)	Sensitivity, % (95% CI)	Specificity, % (95% CI)
Ha [68]	18F-FDG-PET/CT	14 (785)	57 (49–64))	92 (87–95)
Kim [69]	C-11 choline and C-11 acetate PET/CT	10 (282)	66 (54–75)	89 (76–95)
Soubra [70]	18F-FDG-PET/CT	Single-center (78)	56 (29–80)	98 (91–100)
Woo [71]	MRI	24 (2928)	56 (42–69) (per-patient)	94 (90–96) (per patient)
			57 (29–82) (per-lymph node	97 (94–98) (per-lymph node)
